# Carbon-Doped TiO_2_ Activated by X-Ray Irradiation for the Generation of Reactive Oxygen Species to Enhance Photodynamic Therapy in Tumor Treatment

**DOI:** 10.3390/ijms20092072

**Published:** 2019-04-26

**Authors:** Chun-Chen Yang, Min-Hsiung Tsai, Keng-Yuan Li, Chun-Han Hou, Feng-Huei Lin

**Affiliations:** 1 Department of Materials Science and Engineering, National Taiwan University, Taipei 10617, Taiwan; d03527007@ntu.edu.tw; 2 Institute of Biomedical Engineering, National Taiwan University, Taipei 10617, Taiwan; kevininsight99@gmail.com (M.-H.T.); plee0306@hotmail.com (K.-Y.L.); 3 Department of Orthopedic Surgery, National Taiwan University, Taipei 10617, Taiwan; 4 Institute of Biomedical Engineering and Nanomedicine, National Health Research Institutes, Miaoli 35053, Taiwan

**Keywords:** photodynamic therapy, carbon-doped TiO_2_, X-ray, ROS

## Abstract

Traditional photodynamic therapy (PDT) is limited by the penetration depth of visible light. Although the light source has been changed to near infrared, infrared light is unable to overcome the penetration barrier and it is only effective at the surface of the tumors. In this study, we used X-ray as a light source for deep-seated tumor treatment. A particle with a narrow band gap when exposed to soft X-rays would produce reactive oxygen species (ROS) to kill tumor cell, with less damage to the normal tissues. Anatase TiO_2_ has been studied as a photosensitizer in PDT. In the experiment, C was doped into the anatase lattice at an optimum atomic ratio to make the band gap narrower, which would be activated by X-ray to produce more ROS and kill tumor cells under stress. The results showed that the synthesized TiO_2_:C particles were identified as crystal structures of anatase. The synthesized particles could be activated effectively by soft X-rays to produce ROS, to degrade methylene blue by up to 30.4%. Once TiO_2_:C was activated by X-ray irradiation, the death rate of A549 cells in in vitro testing was as high as 16.57%, on day 2. In the animal study, the tumor size gradually decreased after treatment with TiO_2_:C and exposure to X-rays on day 0 and day 8. On day 14, the tumor declined to nearly half of its initial volume, while the tumor in the control group was twice its initial volume. After the animal was sacrificed, blood, and major organs were harvested for further analysis and examination, with data fully supporting the safety of the treatment. Based on the results of the study, we believe that TiO_2_:C when exposed to X-rays could overcome the limitation of penetration depth and could improve PDT effects by inhibiting tumor growth effectively and safely, in vivo.

## 1. Introduction

In clinical applications, photodynamic therapy (PDT) has been a common method of tumor lesion treatment, where a photosensitizer is excited by light of specific wavelengths to induce the formation of reactive oxygen species (ROS), selectively directing tumor cells towards death [1,2]. When the photosensitizers are activated by the light of a specific wavelength, energy is transferred to the oxygen molecules to generate ROS [3]. ROS, including peroxides (•O_2_^2−^), superoxide ions (•O_2_^−^), hydroxyl radicals (•OH), and singlet oxygen (^1^O_2_), can react with unsaturated lipids and nucleic acids to generate oxidative stress, as well as to cause DNA damage and cellular damage of lipids, cholesterol, and proteins on cell membranes [4]. 

However, the light penetration depth, limits the therapeutic efficacy of PDT. Most absorbance bands of the photosensitizers located in the range of visible light, result in penetration depths of less than 1 cm [5]. Although the use of two-photon excitation of nanoparticle-based photosensitizers activated by near-infrared red light could improve the penetration depth to 3 cm, it can only treat the surface of cancer cells and cannot be effectively used for deep-seated tumors [6]. In order to overcome this limitation, Chen et al. proposed luminol, which is a chemical exhibiting chemo-luminescence, to be used as the in situ light source to activate 5-aminolevulinic acid (5-ALA), and meso-tetraphenylporphyrin in 5-ALA mediated the photodynamic treatment of Caco-2 cells. However, the excitation was indirect and less effective because of the low intensity [7]. 

Ionizing radiations, such as X-rays or gamma rays, could be the alternative light sources that enable deeper penetration (8–14 cm) in tissues with optimized sensitizers [8,9,10]. X-rays might interact with molecules to generate free radicals and ROS, which can damage DNA or cellular organelles [11,12]. Nevertheless, the X-ray dose that a patient can receive is limited. For instance, the maximum dose for individual members of the public from a licensed operation must not exceed 1 mSv/year. The limit is 15 mSv/year for the lens of the eye and 50 mSv/year for the skin [13]. This prohibits repeated irradiation at the same site, because the irradiation might extend to normal tissues. Therefore, the tolerance dose of irradiation on normal tissues has become a concern. 

TiO_2_ has been used as a photosensitizer in PDT because of its high photocatalytic activity, low toxicity, great biocompatibility, and chemical stability [14]. Among other crystallographic phases, anatase exhibits the highest photocatalytic activities because of its longer charge-carrier lifetime and low recombination rate of photo-generated charge-carriers [15]. In short, anatase is more suitable to be used as a photosensitizer, compared to rutile and brookite. Although TiO_2_ has been used as a photosensitizer in PDT for cancer therapy, the result has not been very promising. Thus, we tried to dope certain elements into the anatase lattice to narrow the band gap effectively and activate the doped anatase with low-dose X-rays, to reduce irradiation related risks.

To consider the factors of charge-carrier recombination and high-temperature stability, non-metal ions instead of metal ions were selected to be added into the lattice sites of anatase [16,17,18]. Among these, carbon (C) has been proposed to be doped into anatase to shorten the band gap [19]. As known, C has a higher energy in the p orbital than oxygen (O). The substitution of C for O would cause the formation of C 2p impurity states that would have a higher energy level to the valence band and result in a band gap narrowing. We hypothesized that the substitution would cause the anatase lattice to be activated by X-ray much more easily and generate more ROS for tumor 

## 2. Results 

The specific aim of this study was to synthesize carbon-doped TiO_2_ (TiO_2_:C) to act as the photosensitizer in low-dose X-ray-induced PDT to overcome the limitations of penetration depth and radiation dose. The particles were synthesized by the sol–gel method. X-ray diffraction, field-emission scanning electron microscopy, transmission electron microscopy, energy dispersive X-ray spectroscopy, and X-ray photoelectron spectroscopy were performed for crystal structure identification, surface morphology observation, lattice imaging, composition analysis, and surface chemical analysis, respectively. The degradation of methylene blue (MB) was evaluated to examine the generation of ROS [20,21]. The adenocarcinoma human alveolar basal epithelial cell line (A549) was used for in vitro and in vivo studies, to evaluate the effect of the synthesized TiO_2_:C in X-ray-induced PDT. The in vitro testing of the synthesized TiO_2_:C in X-ray-induced PDT was analyzed by lactate dehydrogenase (LDH) and WST-1 assays and then further assessed by live/dead staining. Tumor-induced BALB/c nude mice were used to check the efficacy and efficiency of the designed photosensitizer in in vivo PDT treatment. A549 cells were injected into mouse thighs subcutaneously, to develop tumor lesions as an animal model. The tumor size was measured to examine the therapeutic result of the TiO_2_:C in X-ray-induced PDT. The blood/serum analysis and H-and-E examination were used for the safety assessment of the mice. The results and findings in the study were collected and further analyzed to prove that the developed TiO_2_:C could be the most promising photosensitizer in the X-ray-induced PDT for clinical use. 

### 2.1. Material Characterization

**Figure 1:**
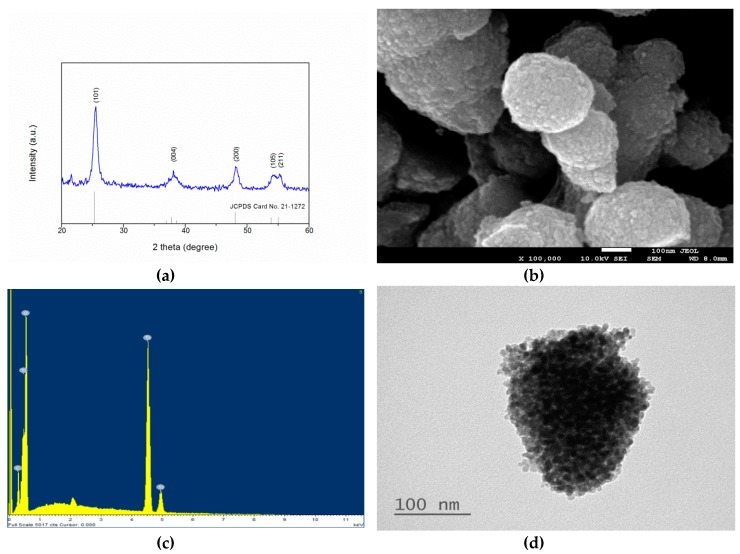
Characterization of the synthesized TiO_2_:C nanoparticles. (**a**) XRD pattern compared with the underlined reference of Joint Committee on Powder Diffraction Standards (JCPDS) card no. 21–1272 for anatase; (**b**) SEM image; (**c**) energy-dispersive X-ray spectroscopy (EDX) spectrum; and (**d**) TEM image.

Figure 1a shows the XRD pattern of TiO_2_:C. The 2-theta at 25.30°, 38.00°, 48.00°, 54.35°, and 55.10° corresponded to (101), (004), (112), (200), (105), and (211), respectively. The peaks and relative intensities of the synthesized TiO_2_:C fully matched the Joint Committee on Powder Diffraction Standards (JCPDS) card No. 21–1272 for anatase TiO_2_. The surface morphology of TiO_2_:C observed with SEM is shown in Figure 1b. TiO_2_:C was synthesized as uniform, spheroid-like aggregated particles with an average diameter of approximately 300 nm. The result of the energy-dispersive X-ray spectroscopy (EDX) analysis (Figure 1c) showed that the major elements in the developed TiO_2_:C were Ti, O, and C. The atomic ratio of C/Ti was 0.025. TEM imaging of the synthesized TiO_2_:C is shown in Figure 1d, where the particle was aggregated by thousands of nano-sized grains. The pores were the spaces left between the particles. The diameter of the nano-grain was approximately 10 nm on average. 

**Figure 2:**
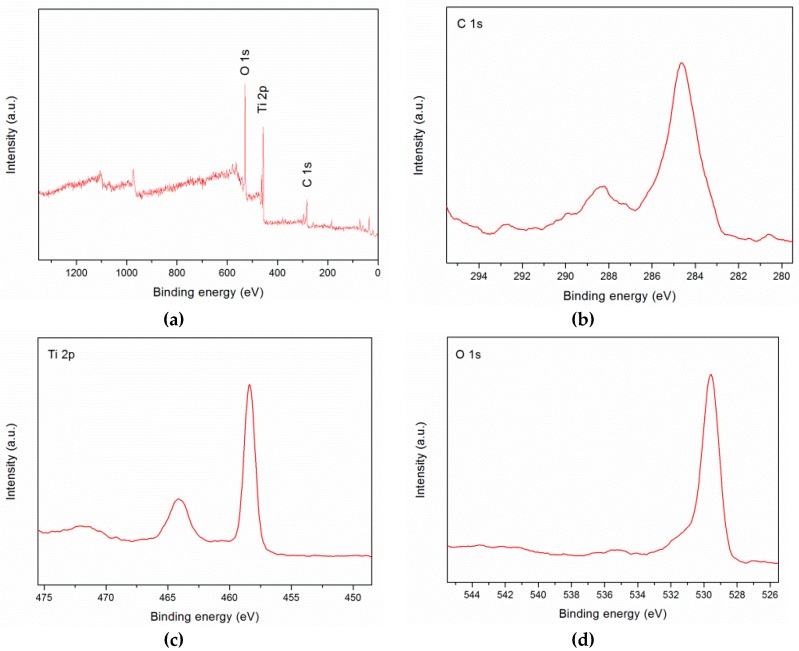
X-ray photoelectron spectroscopy (XPS) analysis of the synthesized nanoparticles. (**a**) General XPS spectrum; (**b**) de-convoluted XPS spectrum of C 1s; (**c**) de-convoluted XPS spectrum of Ti 2p; and (**d**) de-convoluted XPS spectrum of O 1s.

Figure 2a shows the X-ray photoelectron spectroscopy (XPS) spectrum, where C 1s, Ti 2p, and O 1s could be observed at 284, 457, and 529 eV, respectively. The peak at 284 eV is the combination of the three peaks, as shown in Figure 2b, where the peaks located at 281.5, 284.65, and 288.4 eV are the Ti-C bond, adventitious carbon C-C bond, and C-O bond, respectively. These data revealed that carbon may have substituted some of the lattice titanium atoms and formed a Ti-O-C structure. If the peak of Ti 2p was further scanned, the peak could be split into two binding energies at 458.4 and 464.1 eV that could be identified as Ti 2p_3/2_ and Ti 2p_1/2_, respectively, as shown in Figure 2c. The peak at 529.6 eV corresponds to the lattice O bound to Ti^4+^, an O-Ti^4+^ covalent bond (Figure 2d). 

### 2.2. ROS Generation of TiO_2_:C under X-ray Irradiation

**Figure 3:**
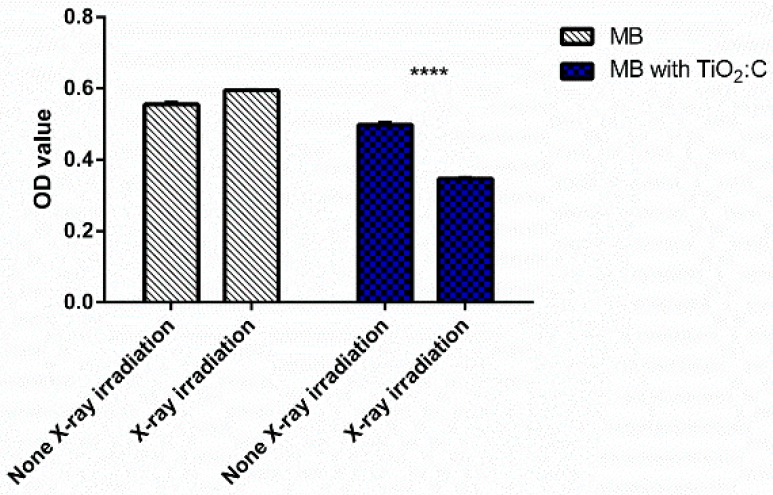
The generation of ROS of the synthesized TiO_2_:C under X-ray irradiation determined by the degradation of methylene blue. The measurement was performed at a wavelength of 664 nm. The concentration of TiO_2_:C particles and MB were 1 mg/mL and 10 ppm, respectively. The exposure of X-ray irradiation is 100 s (*t*-test, mean ± SD, *n* = 6, ****: *p*-value < 0.0001).

Figure 3 shows that MB solution without TiO_2_:C particle addition was not degraded by X-ray irradiation. When 1 mg/mL TiO_2_:C particles were added into the MB solution and exposed to X-rays, MB was degraded by up to 30.40% compared to that without X-ray exposure. This result indicated the significant generation of ROS from TiO_2_:C when exposed to X-ray irradiation for 100 s. 

### 2.3. Efficacy of TiO_2_:C in Killing A549 Cells under X-ray Irradiation In Vitro

**Figure 4:**
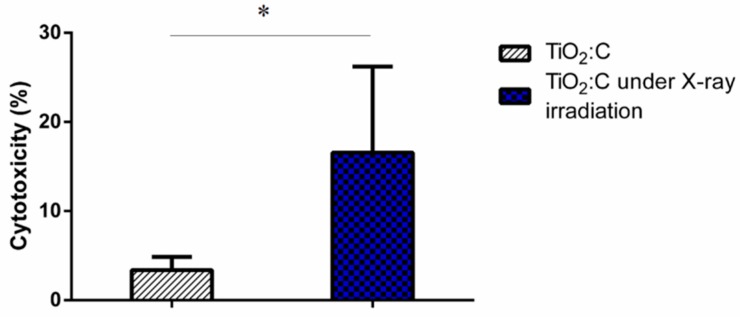
The photodynamic therapy (PDT) efficacy of TiO_2_:C exposed to X-ray irradiation in terms of toxicity by LDH assay (*t*-test, mean ± SD, *n* = 6, *: *p*-value < 0.05).

The in vitro PDT effects of TiO_2_:C activated by X-ray irradiation was evaluated using LDH assay with A549 lung cancer cells (Figure 4). Without X-ray irradiation, the developed TiO_2_:C showed no significant toxicity (as low as 3.38 ± 1.48% on day 2). When TiO_2_:C was applied with X-ray irradiation, the death rate of A549 cells increased to 16.57 ± 9.63%. 

**Figure 5:**
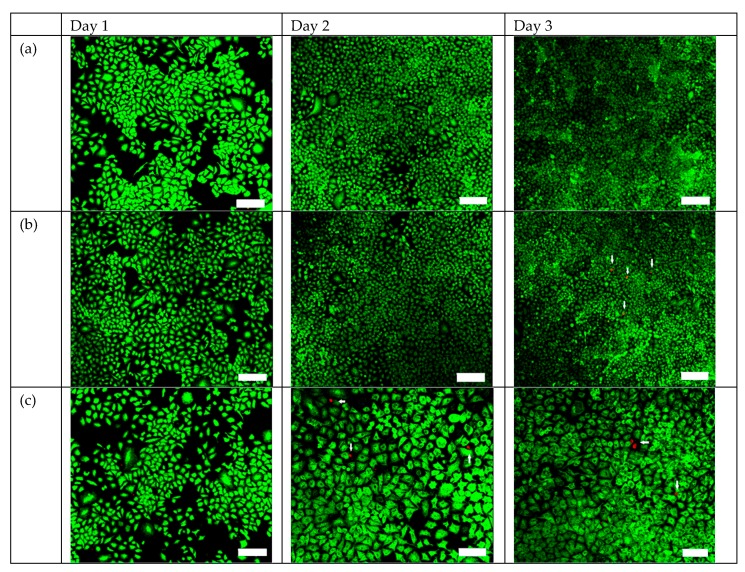

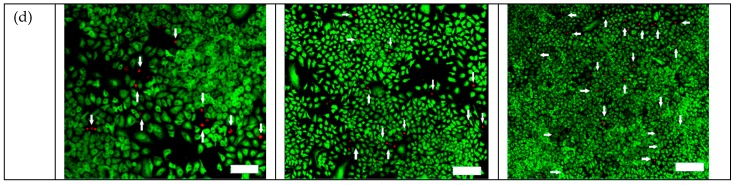
The PDT efficacy of TiO_2_:C exposed to X-ray irradiation, evaluated by live/dead staining of (**a**) control group; (**b**) X-ray-treated group; (**c**) TiO_2_:C-treated group; and (**d**) TiO_2_:C-X-ray group (scale bars: 200 μm), where green represents living cells and red represents dead cells.

The results of live/dead assay are shown in Figure 5 to evaluate cell viability in TiO_2_:C during X-ray irradiation. From day 1 to day 3, a large number of live A549 cells (green) with few dead cells (red) appeared in the control group (Figure 5a). The X-ray-exposed group (Figure 5b) and TiO_2_:C group (Figure 5c) also revealed very few dead cells, which were in agreement with the biocompatibility cell viability results. Figure 5d displays a higher level of dead cells, indicating that the cell viability decreased and that the potential risk of cellular death was caused by TiO_2_:C when activated by X-ray irradiation.

### 2.4. In Vivo Anti-Tumor Efficacy of TiO_2_:C under X-ray Irradiation (TiO_2_:C-X-ray)

**Figure 6:**
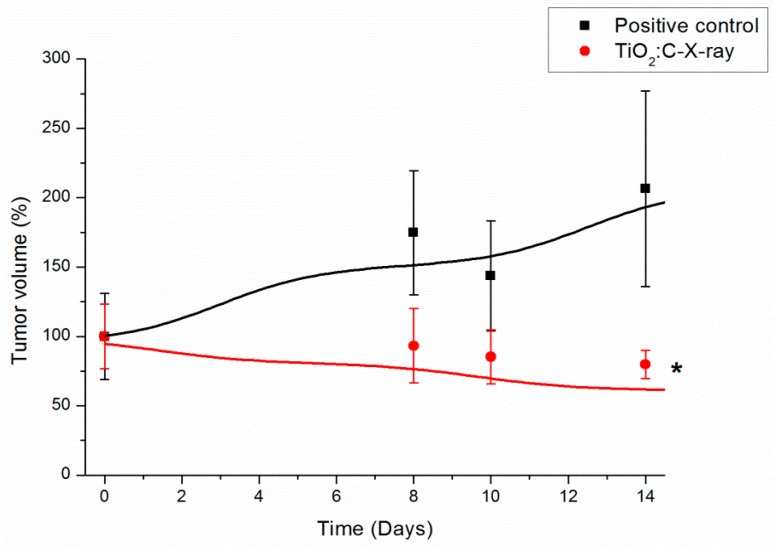
Tumor size in BALB/c nude mice injected with TiO_2_:C and subjected to X-ray irradiation (*t*-test, mean ± SD, *n* = 4, *: *p*-value < 0.05).

After the PDT effect of TiO_2_:C under X-ray irradiation was proven to kill A549 cancer cells in vitro, in vivo study further proceeded to prove the PDT effect of the developed particles under X-ray irradiation, as shown in Figure 6. The anti-tumor efficacy of TiO_2_:C under X-ray irradiation was evaluated using male BALB/c nude mice, subcutaneously injected with A549 cells, where TiO_2_:C particles were delivered into mice in only one shot, during the entire experiment, and then treated with X-ray irradiation twice, on day 0 and day 8. As shown in Figure 6, the tumor volume in the control group at day 14 was up to twice that of the initial volume at day 0. On the contrary, at day 14, the tumor volume in the TiO_2_:C-X-ray group gradually decreased to nearly half of the initial volume.

### 2.5. Blood/Serum Analysis and Hematoxylin and Eosin (H-and-E) Examination

**Figure 7:**
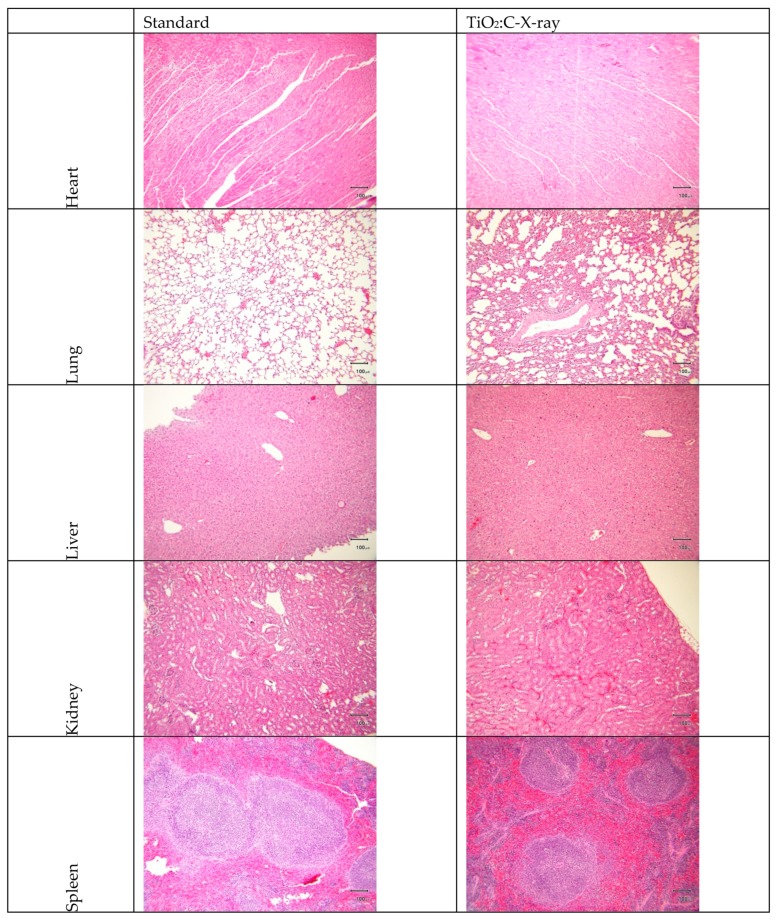
Hematoxylin and Eosin (H-and-E)-stained slices of major organs harvested from scarified BALB/c nude mice at the end of the animal study and examined under optical microscope (scale bar: 100 μm).

The safety efficacy of TiO_2_:C-X-ray was evaluated by whole blood/serum analysis and hematoxylin and eosin (H-and-E) examination. From blood/serum bio-chemical analysis (Table 1), all parameters of the TiO_2_:C-X-ray group complied with the standard and were in the range of standards of blood bio-chemical analysis (Table 2). The heart, lung, liver, kidney, and spleen were taken from the mice to determine toxicity in the mice that could arise from the in vivo presence of TiO_2_:C and the effect of X-ray irradiation. Figure 7 shows no significant difference between the experimental group and non-treated group, including no observable signs of side effects on normal tissues from H-and-E staining.

## 3. Discussion

TiO_2_:C was successfully synthesized by the modified sol–gel method and was confirmed by the crystalline phase identification, surface morphology observation, and chemical bond analysis (Figure 1). Pluronic F-127 was the amphiphilic triblock copolymers and biodegradable non-ionic surfactant for the production of TiO_2_ with glucose as the carbon source. Due to C doping, the substitution of C for O in TiO_2_ formed C 2p impurity states that were higher than the valence band of TiO_2_. This impurity state also contributed a band-tailing effect, resulting in a band gap narrowing. The results showed that the band gap reduced from 3.2 eV (TiO_2_-anatase) to approximately 3.07 eV, which could be easily activated by low-dose X-ray irradiation (Figure S1).

Based on ISO 10993 regulations, the toxicity of TiO_2_:C and its effect on cell viability indicated no potential risk to cells as shown in Figure S2. We also further investigated the effect of ROS generation on TiO_2_:C, under X-ray irradiation. 

As shown in Figure 3, only a negligible decrease in optical density was detected for the TiO_2_:C group without X-ray irradiation, compared to the control group. These results indicated that TiO_2_:C is a safe biomaterial with no significant intracellular ROS generation. TiO_2_:C, when activated by X-rays, generated ROS, such as •OH and •O^2−^, resulting in reduction–oxidation of MB [22,23]. The optical density significantly decreased, due to the degradation of MB, which indicated the increasing levels of ROS. The increase in ROS caused by exposure to X-ray irradiation might be attributed to the oxygen enhancement of radiolysis by ionizing radiation. Low-energy X-rays (<1 MeV) interact with matter via the photoelectric effect [24]. The photoelectric effect occurs when incident photons are absorbed by atoms and the inner-shell electrons are ejected. Free radicals are produced by the interactions between X-rays and the biological molecules present in sensitizers [25,26]. ROS can readily react with all types of biomolecules and damage cellular structure. Among the biological targets, the ones that are most vulnerable to oxidative damage are proteinaceous enzymes, lipidic membranes, and DNA. By targeting these biomolecules, ROS might directly harm their main sites of production (mitochondria) and influence cellular viability via mitochondria-dependent or independent pathways.

LDH, a stable cytosolic enzyme that is only released when the cell membrane is damaged, was used to evaluate cell toxicity. TiO_2_:C activated by X-rays, while in contact with cell membranes, enhanced the production of ROS, which caused oxidative damage, and demonstrated peroxidation of membrane lipids, resulting in a loss of membrane integrity [8,27,28]. The loss of membrane integrity is one of the confirmed indicators of cell death [29]. Plasma membrane damage can be viewed as an indirect effect of ROS generated by X-ray-activated TiO_2_:C near cells, causing toxicity (Figure 4). The toxicity caused by the increase in ROS level was involved in different molecular cell death mechanisms (necrosis and apoptosis) (Figure 5) [30,31]. ROS formation triggers lipid peroxidation, which adversely affects the oxidative phosphorylation system and mitochondrial membrane potential [32,33]. The products of lipid peroxidation will interact with lipids present in the mitochondrial bilayer, impairing their function and resulting in the opening of the mitochondrial permeability transition pore (MPTP) [34]. The MPTP is a major player in apoptosis and necrosis as its induction under various conditions triggers cell death via either of the mechanisms. Increase in the intracellular levels of ROS triggers the opening of the MPTP and a subsequent decrease in the mitochondrial membrane potential. The opening of the MPTP leads to the release of mitochondrial intermembrane proteins, such as cytochrome c from the mitochondria and into the cytosol [35]. The release of cytochrome c into the cytosol results in the formation of the apoptosome complex by its interaction with the apoptotic protease-activating factor 1. In this caspase-dependent signaling pathway, the apoptosome complex recruits procaspase 9, which induces the activation of the downstream effectors, caspase 3 and caspase 7, leading to apoptosis [36]. These results were in agreement with the in vitro and in vivo tumor size observations (Figure 6) that TiO_2_:C activated by X-rays could be a potent method to produce numerous ROS, which cause cell death and inhibit tumor growth. TiO_2_:C particles are also non-toxic in the absence of X-rays. Meanwhile, they are highly biodegradable and can be efficiently cleared from the hosts after treatment, causing no side effects to normal tissues (Figure 7). These results are consistent with the in vitro and in vivo observations that TiO_2_:C activated by X-rays could be a promising approach for further clinical use.

## 4. Materials and Methods

### 4.1. Synthesis of TiO_2_:C Particles

Titanium (IV) isopropoxide (TIP; Ti(OCH(CH_3_)_2_)_4_, purity >97%, batch No. BCBN5487V, Sigma-Aldrich, St. Louis, USA) was the precursor of TiO_2_, and d-(+)-glucose (C_6_H_12_O_6_, purity ≥99.5%, batch No. 129H0342, Sigma-Aldrich) was used as the carbon source. Two grams of Pluronic F-127 (batch No. BCBL7068V, Sigma-Aldrich) was dissolved in 40 mL of absolute ethanol with magnetic stirring, and 5 mL of TIP was added and dissolved in the mixture. Three grams of glucose were slowly added into the solution and stirred until it dissolved completely. The solution was kept in a static state, for precipitation. The precipitate was collected by centrifugation at 5,000 rpm, washed three times with absolute ethanol, and dried at 100 °C for 20 min. The obtained powder was calcined at 400 °C for 2 h.

### 4.2. Material Characterization 

The crystal structure of the synthesized TiO_2_:C was identified by X-ray diffractometry (XRD; Philips, 1830/Mac, Amsterdam, Netherlands) with CuKα radiation (λ = 0.15406 nm). The samples were scanned from 20° to 60° at a speed of 2°/min at 30 kV and 15 mA. The surface morphologies were observed with a field-emission scanning electron microscope (FESEM; JEOL, JSM-7600F, Peabody, MA, USA) at 10 kV and 30 mA. The chemical composition of the samples was analyzed with energy-dispersive X-ray spectroscopy (EDX; JEOL, JSM-7600F). Lattice images were obtained and morphology was analyzed by transmission electron microscopy (TEM; JEOL, JEM-1200EX II) at 120 kV. The synthesized TiO_2_:C was dispersed in double-distilled H_2_O with an ultrasonic bath, dropped on 400-mesh copper grids, and dried in air. The specimens were mounted on a sample holder for further analysis in a high-vacuum chamber. X-ray photoelectron spectroscopy (XPS; Thermo Scientific, Theta Probe, Waltham, MA, USA) was used for surface chemical analysis.

### 4.3. Degradation of MB for ROS Detection

In order to evaluate ROS production of the synthesized TiO_2_:C under X-ray irradiation, the degradation of MB was analyzed by UV-Vis spectroscopy. X-rays were generated by an X-ray apparatus (POYE, PX-80M, New Taipei, Taiwan) at 80 kV and 10 mA at a dose rate of 0.08 Gy/min for 100 s. Ten micrograms of TiO_2_:C was dispersed in 10 mL of aqueous solution of 10 ppm MB. The degradation of MB was evaluated by an enzyme-linked immunosorbent assay (ELISA) reader at an absorbance of 664 nm.

### 4.4. LDH Assay: In Vitro Testing of the Synthesized TiO_2_:C in X-ray-Induced PDT 

A549 cells (3 × 10^3^ cells/well) were seeded on a 96-well plate and then incubated at 37 °C in a 5% CO_2_ atmosphere [37]. The synthesized TiO_2_:C (1 mg/mL) in 100 μL of medium was added to each well, one day after seeding. Cells were irradiated with X-rays generated at 80 kV and 10 mA, at a dose rate of 0.08 Gy/min for 100 s. Lysis buffer (100 μL) was added to each well in the lysis group and incubated at 37 °C in a 5% CO_2_ atmosphere for 45 min. The culture medium was collected for the LDH assay, and 50 μL of culture medium was reacted with 50 μL of LDH reagent and kept in the dark at 25 °C for 30 min. After that, 50 μL of stop solution was added to each well to stop the reaction, and the cell death rate was evaluated using an ELISA reader, by measuring the absorbance of 490 nm. F12K medium (SLBQ791VV, Sigma-Aldrich) supplemented with 10% fetal bovine serum and 1% antibiotics-antimycotics was used for A549 cell culture. Each experiment was repeated six times.

### 4.5. Live/Dead Staining: In Vitro Testing of the Synthesized TiO_2_:C in X-ray-Induced PDT

A549 cells were seeded in 3.5-cm culture dish at a density of 8.5 × 10^4^ cells/mL and cultured for one day. The synthesized TiO_2_:C (1 mg/mL) in 100 μL of medium was added to each culture dish. As described in Section 2.3, cells were irradiated with X-ray generated at 80 kV and 10 mA at a dose rate of 0.08 Gy/min for 100 s. All dishes were incubated at 37 °C in a 5% CO_2_ atmosphere for one day. In the following three days, l mL of live/dead reagent (1387211, Invitrogen, Carlsbad, CA, USA) prepared with phosphate-buffered saline (PBS) was added into each dish and kept in the dark, in a static state for 30 min. The cells were then examined under a confocal microscope (Olympus, BX51, Tokyo, Japan). 

### 4.6. In Vivo Evaluation of the PDT Effect of the Synthesized TiO_2_:C under X-ray Irradiation 

A549 cells (5 × 10^5^) suspended in 100 μL of PBS were subcutaneously injected into the thighs of five-week-old male BALB/c nude mice, which were maintained in cages by normal feeding until the tumors grew to a volume of 50 mm^3^. The mice were divided into two groups—(1) the positive control group was injected with only PBS at day 0 and day 8, without X-ray irradiation; and (2) the experimental group, abbreviated as TiO_2_:C-X-ray, was subjected to intratumor injection of TiO_2_:C nanoparticles at day 0 and day 8, followed by X-ray irradiation. 

### 4.7. In Vivo Safety Testing of the Synthesized TiO_2_:C under X-ray Irradiation

At the end of the animal study in Section 4.6, all mice were sacrificed. The blood sample and internal organs were harvested for later analysis and examination as follows. Major organs were harvested and fixed with 10% formaldehyde. Fixed tissues were embedded in paraffin, sectioned into slices with a thickness of 5 μm, and stained with H-and-E for histological examination. Whole blood was collected by cardiac puncture, sampled into a tube with 7.5% EDTA solution, and sent to the Animal Center of National Taiwan University for further analysis. Blood/serum bio-chemical analysis involved renal function testing, liver function testing, and electrolyte examination. Blood urine nitrogen (BUN) and creatinine (CREA) were used to assess renal function. Alanine aminotransferase (ALT), aspartate aminotransferase (AST), and albumin (ALB) were the measures of liver function used. Calcium (Ca) and inorganic phosphate (IP) were measured as an assessment of electrolytes. Blood bio-chemical analysis comprised blood routine and white blood cell classification. All tests involving animals were carried out at the Animal Center of National Taiwan University. All animal experiments were performed according to the guidelines determined by the Animal Center of National Taiwan University for Animal Welfare (project identification code 20170269, 6 February 2017).

### 4.8. Statistical Analysis

The data were collected and expressed as mean ± standard deviation (SD) for the control and experimental groups. Statistical analysis was performed using *t*-test. GraphPad Prism 6 (GraphPad Software, San Diego, CA, USA) was used for analysis in this study. *p*-value < 0.05 was considered statistically significant.

## 5. Conclusions

We have developed TiO_2_:C particles activated by X-rays as a promising approach to produce ROS and enhance PDT. The TiO_2_:C particles were synthesized at the nano-scale in the anatase phase. The band gap was effectively reduced by doping with C, which could be easily activated by X-rays to generate ROS. The cell viability and toxicity of TiO_2_:C evaluated by WST-1 and LDH assays showed no intracellular toxicity of TiO_2_:C to normal cells. The toxicity was increased when TiO_2_:C was activated by X-rays, and cell death was induced via necrotic or apoptotic pathways. The result was consistent with ROS testing by the degradation of MB. The in vivo study also showed tumor growth inhibition based on the cell death mechanism, induced by ROS. Safety assessment by blood/serum analysis and H-and-E examination indicated that TiO_2_:C is not toxic and caused no side effects to the normal tissues. This study provided a novel therapeutic approach as an alternative deep-seated tumor/cancer treatment in the near future.

## Figures and Tables

**Table 1:** Bio-chemical analysis of the blood/serum samples from the TiO_2_:C-X-ray group.

Parameter	Unit	Standard	TiO_2_:C-X-ray
BUN	mg/dL	39.15 ± 12.16	30.81
CREA	mg/dL	<0.2 (0.1)	<0.2 (0.1)
ALT(SGPT)	U/L	33.5 ± 2.12	30.00
AST(SGOT)	U/L	80 ± 13.22	71.50
ALB	g/L	28.3 ± 3.89	25.55
Ca	mmol/L	2.23 ± 0.11	2.29
IP	mg/dL	6.29 ± 2.22	6.70

**Table 2:** Bio-chemical analysis of blood samples from the TiO_2_:C-X-ray group.

Parameter	Unit	Standard	TiO_2_:C-X-ray
RBC	M/μL	8.64 ± 0.48	8.46
HGB	g/dL	13.5 ± 0.69	13.20
HCT	%	41.3 ± 2.23	41.50
MCV	fL	45.65 ± 3.04	47.53
MCH	pg	14.31 ± 1.86	15.55
MCHC	g/dL	31.34 ± 1.9	31.72
WBC	K/μL	2.81 ± 1.49	2.30
NEUT	K/μL	2.14 ± 0.32	1.55
LYMPH	K/μL	1.62 ± 0.44	1.20
MONO	K/μL	0.13 ± 0.02	0.24
PLT	K/μL	767 ± 208.75	780.00
MPV	fL	6.1 ± 0.43	6.20

## Supplementary Materials

Supplementary materials can be found at https://www.mdpi.com/1422-0067/20/9/2072/s1.

## Abbreviations

PDT	photodynamic therapy
ROS	reactive oxygen species
A549 cells	adenocarcinomic human alveolar basal epithelial cells
DNA	deoxyribonucleic acid
5 ALA	5-Aminolevulinic Acid
Caco-2	heterogeneous human epithelial colorectal adenocarcinoma cell line
MB	methylene blue
A549	adenocarcinoma human alveolar basal epithelial cell line
LDH	lactate dehydrogenase
TIP	titanium (IV) isopropoxide
PBS	phosphate buffered saline
H-and-E	hematoxylin and eosin
EDTA	ethylenediaminetetraacetic acid
BUN	blood urine nitrogen
CREA	creatinine
ALT	alanine aminotransferase
AST	aspartate aminotransferase
ALB	albumin
Ca	calcium
IP	inorganic phosphate
MPTP	mitochondrial permeability transition pore
