# Supplementary material for: Carbon-Doped TiO2 Activated by X-Ray Irradiation for the Generation of Reactive Oxygen Species to Enhance Photodynamic Therapy in Tumor Treatment
Source: Int J Mol Sci. 2019 Apr 26;20(9):2072. doi: 10.3390/ijms20092072 (PMC6540153; doi:10.3390/ijms20092072)
Supplement: Supplementary file 1 [file ijms-20-02072-s001.pdf]

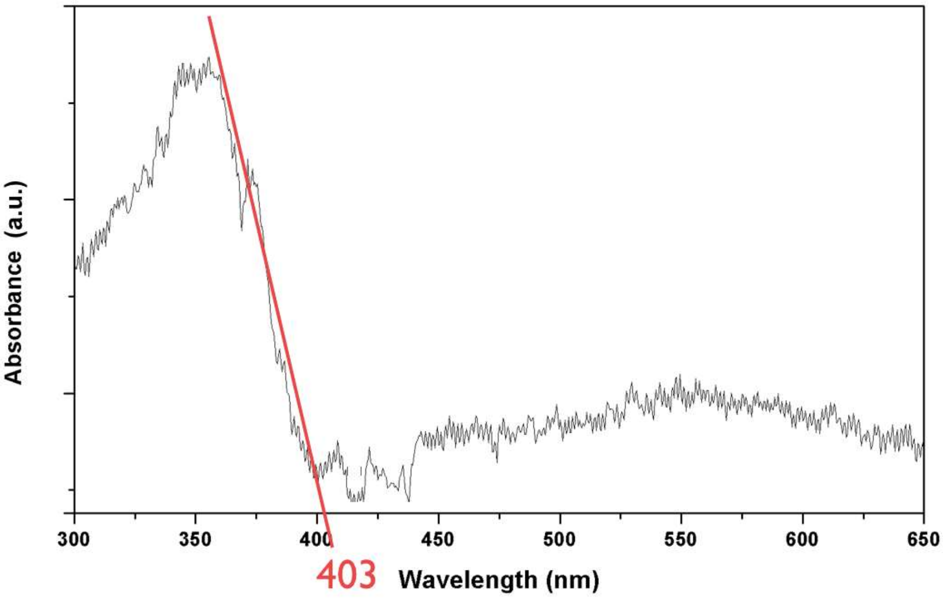

Figure S1. Bandgap of TiO_2:C under X-ray irradiation from UV–Vis spectroscopy.

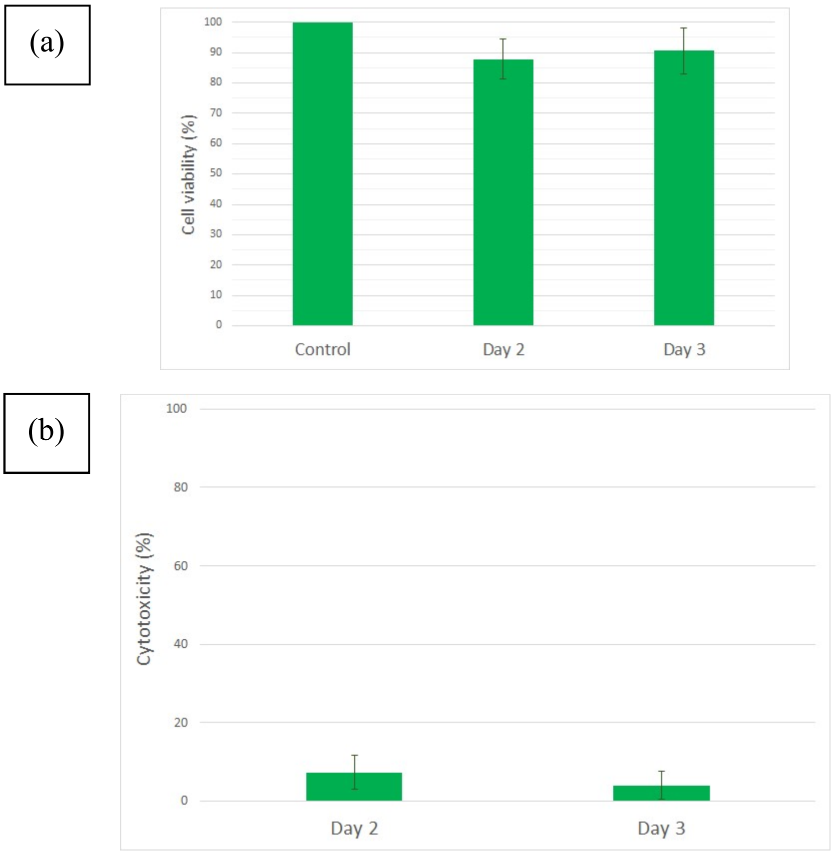

Figure S2. (**a**) Cell viability and (**b**) cytotoxicity of the synthesized TiO_2:C at day 2 and day 3.
